# Supplementary material for: Phonon-mediated room-temperature quantum Hall transport in graphene
Source: Nat Commun. 2023 Jan 19;14:318. doi: 10.1038/s41467-023-35986-3 (PMC9852447; doi:10.1038/s41467-023-35986-3)
Supplement: Supplementary file 1 — Supplementary Information [file 41467_2023_35986_MOESM1_ESM.pdf]

Supplementary Information

Phonon-mediated room-temperature quantum Hall transport in graphene

Daniel Vaquero[1,†], Vito Clericò[1,†], Michael Schmitz[2,3], Juan Antonio Delgado-Notario[1,4], Adrian Martín-Ramos[1], Juan Salvador-Sánchez[1], Claudius S. A. Müller[5,6], Km Rubi[5,6], Kenji Watanabe[7], Takashi Taniguchi[8], Bernd Beschoten[2], Christoph Stampfer[2,3], Enrique Diez[1], Mikhail I. Katsnelson[6], Uli Zeitler[5,6], Steffen Wiedmann[5,6], Sergio Pezzini[9,*]

[1]*Nanotechnology Group, USAL–Nanolab, Universidad de Salamanca, E-37008 Salamanca, Spain.*

[2]*JARA-FIT and 2nd Institute of Physics, RWTH Aachen University, 52074 Aachen, Germany.*

[3]*Peter Grünberg Institute (PGI-9), Forschungszentrum Jülich, 52425 Jülich, Germany.*

[4]*CENTERA Laboratories, Institute of High Pressure Physics, Polish Academy of Sciences, 29/37 Sokołowska Str, Warsaw, Poland.*

[5]*High Field Magnet Laboratory (HFML-EMFL), Radboud University, Toernooiveld 7, 6525 ED Nijmegen, The Netherlands.*

[6]*Radboud University, Institute for Molecules and Materials, Heyendaalseweg 135, 6525 AJ Nijmegen, The Netherlands.*

[7]*Research Center for Functional Materials, National Institute for Materials Science, 1-1 Namiki Tsukuba, Ibaraki 305-0044, Japan.*

[8]*International Center for Materials Nanoarchitectonics, National Institute for Materials Science, 1-1 Namiki Tsukuba, Ibaraki 305-0044, Japan.*

[9]*NEST, Istituto Nanoscienze-CNR and Scuola Normale Superiore, Piazza San Silvestro 12, 56127 Pisa, Italy.*

[†]*These authors contributed equally to this work*

**email: sergio.pezzini@nano.cnr.it*

Supplementary Note 1: Temperature dependence of the phonon occupation at high fields

According to Ref. [24], the inverse of the electronic magnetic length determines the wave-vectors of phonons contributing to electron-phonon scatterings in magnetic fields. As a consequence, the energy scale $E_{ph} = \hbar \mathrm{v}_s / l_B$ is introduced. Moreover, due to the high energy range of optical phonons (1800–2300 K) leading to small phonon occupation numbers, only acoustic phonons can be considered. In Figure S1, we therefore show the field dependence of E_{ph} (inset), as well as the phonon occupation number at B = 30 T (main panel), for the longitudinal and transverse acoustic phonons in graphene (both included in the calculations of Ref. [24]).

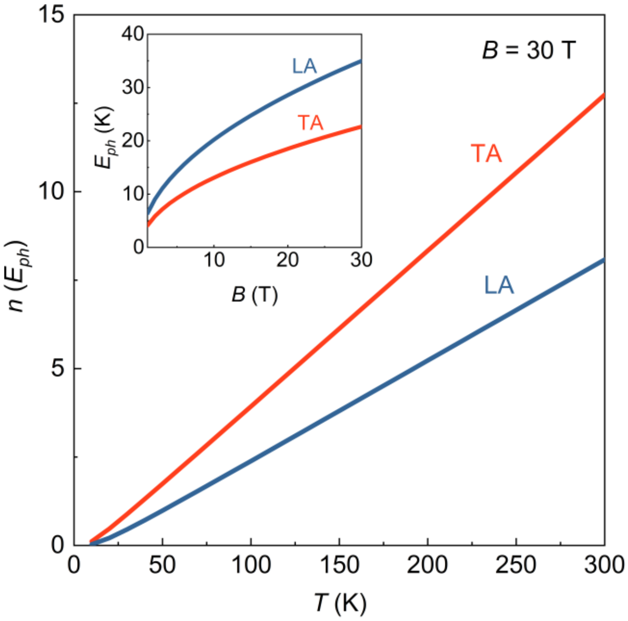

Figure S1 | *B* and *T*-dependent acoustic phonons in graphene. Probability of thermally-excited longitudinal (LA) and transverse (TA) acoustic phonons in graphene, with energy E_{ph} (defined as in the main text) at B = 30 T, calculated according to $n(E_{ph}) = 1/(\exp\left(\frac{E_{ph}}{kT}\right) - 1)$. Inset: field dependence of E_{ph}, calculated using a sound velocity $\mathrm{v}_s = 2.1 \times 10^4$ m/s (1.36×10^4 m/s) for LA (TA) (values from Ref. [36]).

Supplementary Note 2: Zero-field temperature-dependent resistivity

In Figure S2, we show the zero-field resistivity of devices D1-3, measured in temperature and carrier density ranges relevant to our study (T = 170 K - 300 K, $n = 1 \times 10^{12}$ cm^{-2}). For all samples, a clear monotonic increase of the resistivity can be observed, as generally accepted for high-mobility single-layer graphene and attributed to carrier scattering with thermally excited phonons [5-10]. From linear fits to the data, we find slopes within 0.24 Ω/K and 0.45 Ω/K, comparable with Ref. [5] (~0.3 Ω/K at the same carrier density, obtained from the linear fit shown in Figure S2). In accordance with the mobility data discussed in the main text (Figure 2a), we observe that device D3 closely approximates the behaviour of reference data from Ref. [5], indicating analogous sample quality, and dominant e-ph scattering close to RT. The resistivity of devices D1 and D2, as expected from their lower carrier mobility, is offset due to residual disorder scattering adding to the e-ph mechanism.

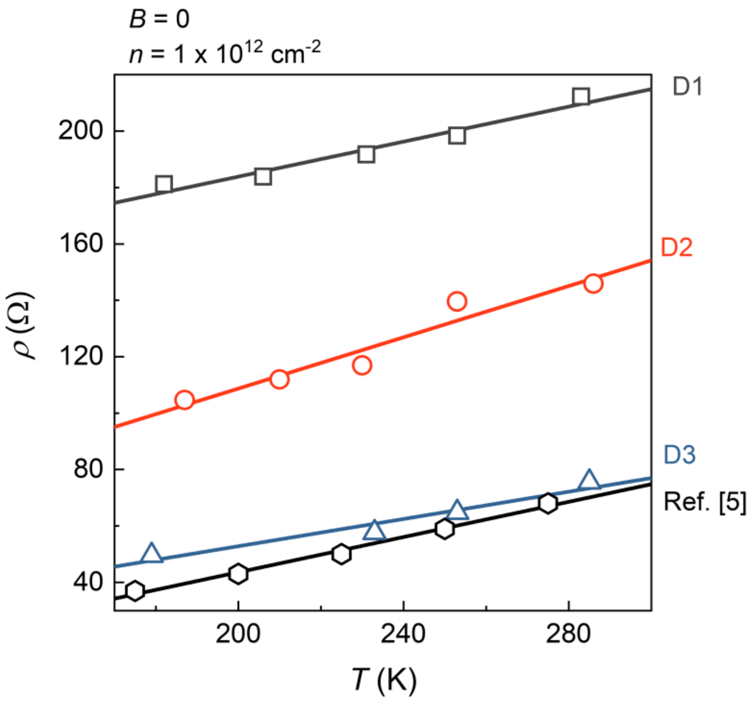

Figure S2 | *T*-dependent resistivity at *B* = 0. Resistivity of samples D1-3 (open symbols), as a function of temperature, measured at $B = 0$ and $n = 1 \times 10^{12}$ cm^{-2}. Reference data from Ref. [5] at the same carrier density are shown as open black hexagons. The continuous lines are linear fits to the data.

Supplementary Note 3: Low-temperature transport at high magnetic fields

As well-established in hBN-encapsulated graphene devices, the application of high magnetic fields promotes correlation-driven phases. Accordingly, both integer QH states outside the half-integer 2, 6, 10 ,… sequence, and fractional QH states are observed at low temperature in our samples (data for sample D2 are shown in Figure S3; see Ref. [31] for sample D4).

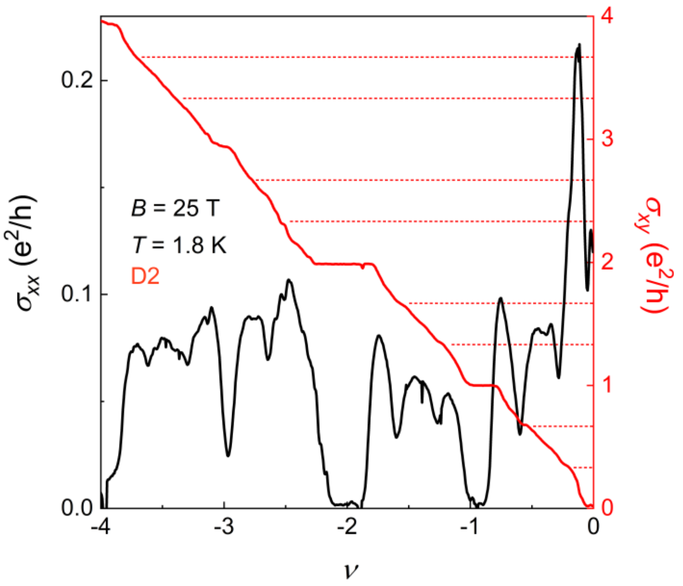

Figure S3 | Fractional quantum Hall effect in sample D2. Longitudinal (black) and Hall conductivity (red) as a function of filling factor, measured in sample D2 at B = 25 T, T =1.8 K. In addition to broken-symmetry integer QH states at ν = -1, -3, -4, fractional QH states at ν = -1/3, -2/3, -4/3, … are clearly resolved. The corresponding fractionally quantized values in σ_{xy} are indicated by the horizontal dashed red lines. In the temperature range relevant to our RT-QH experiments, both the broken-symmetry integer states and the fractional states are suppressed due to their small gap size.

Supplementary Note 4: Phonon-mediated limit at *ν* = -2

Given the particle-hole symmetric electronic structure of graphene, the same behaviour observed at filling factor *ν* = 2 (see main text) should be followed by the activated resistivity at *ν* = -2. In Figure S4 we show that this is indeed the case: data from samples D1-D3 group close to the e-ph limit, with D3 reasonably following the exact theoretical curve (see inset).

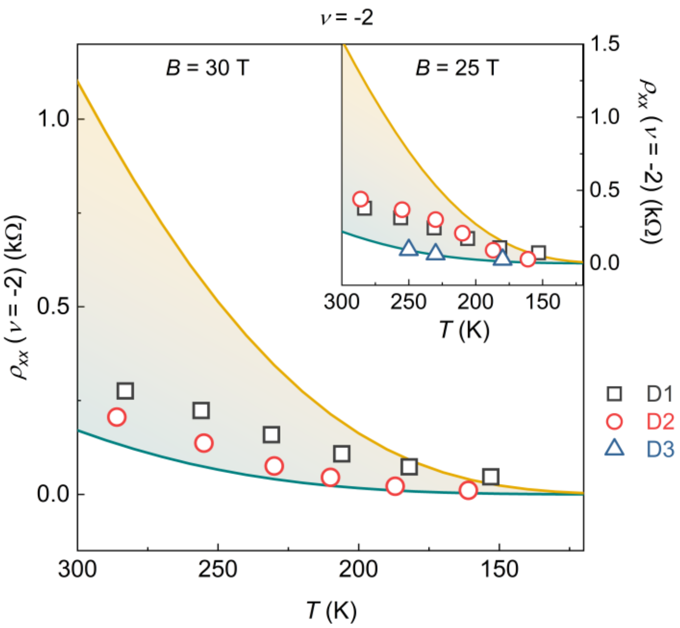

Figure S4 | Temperature-dependent resistivity at *ν* = -2. Activated resistivity at filling factor -2 (hole doping) as a function of temperature, for two different magnetic fields (30 T and 25 T, in the main panel and inset, respectively). The yellow and dark cyan continuous lines are calculations, as in main text Figure 3c.

Supplementary Note 5: Arrhenius plots

Given the 1/T-exponential dependence of the activated conductivity, it is not surprising that an Arrhenius-type behaviour can describe our data. When considering the pre-factor σ_{Arr} (Figure S5e), the clear contrasting behaviour between disordered and clean samples can be appreciated. While data from Ref. [20] approximate the long-range disorder limit ($2e^2/h$ multiplied by the factor 4, accounting for spin and valley degeneracy), our samples show dramatically lower values. As discussed in the manuscript, the magnitude of the pre-factor for sample D3 matches the predictions of Ref. [24], while small corrections need to be considered for D1 and D2, correlating with their lower zero-field mobility.

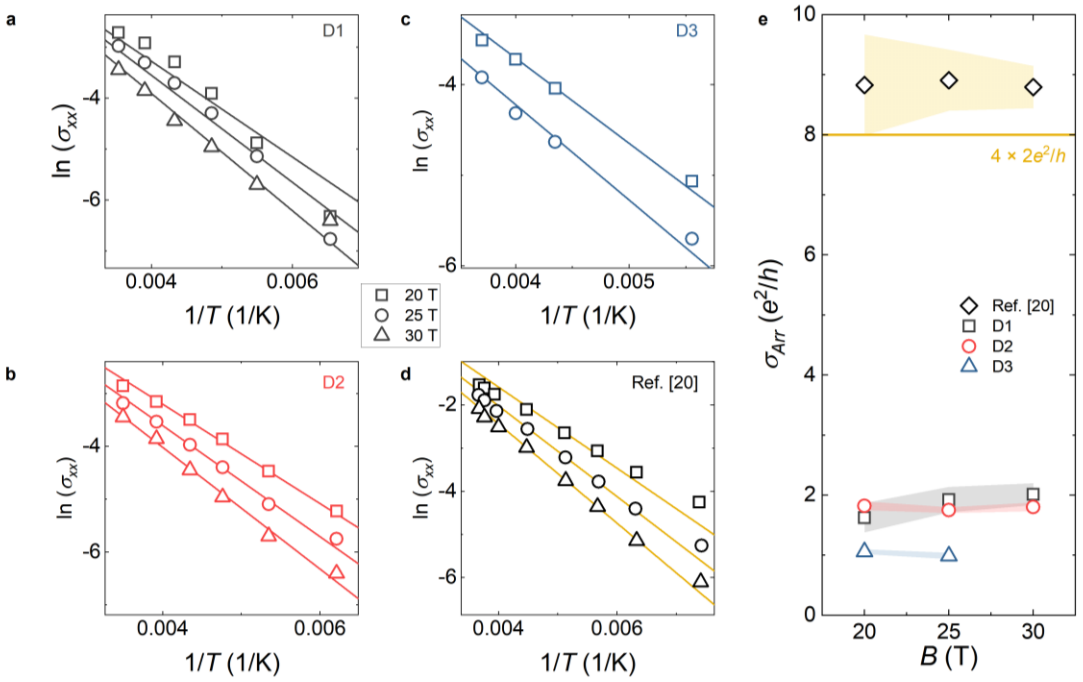

Figure S5 | Arrhenius fits to the activated conductivity. a, Arrhenius fits $\sigma_{xx} = \sigma_{Arr}\exp(-\Delta_{LL}/2kT)$ for sample D1 at different magnetic fields. The activation energy is kept at half of the bare LL gap, following the results of Ref. [20]. **b,** Arrhenius fits for sample D2. **c,** Arrhenius fits for sample D3. **d,** Arrhenius fits on data from Ref. [20]. **e,** Conductivity pre-factors σ_{Arr} from the fits in panels a-d. The shaded areas correspond to ± standard error on the best-fit intercept from linear fits to ln(σ_{xx}) vs 1/T.

Supplementary Note 6: Characteristic field and temperature dependence of the e-ph pre-factor

In this section, we investigate the temperature and field dependence of the e-ph pre-factor form data on samples D1-3. Taking into account the term σ_0 from Ref. [24] and the constant correction σ_D (expressed as resistivity ρ_D in Figure 4 and relative discussion), the activated conductivity of our samples can be expressed as $\sigma_{xx} = (\sigma_0 + \sigma_D)\exp(-\Delta_{LL}/2kT) = (\sigma_N\, \sigma_T\, \sigma_B + \sigma_D)\exp(-\Delta_{LL}/2kT)$, where we name σ_T and σ_B the temperature and field dependent components, respectively. From our data, we can easily extract σ_T and σ_B for each temperature and magnetic field, and compare them with the predictions of Ref. [24], $\sigma_T = T/300$ K and $\sigma_B = (B/10\text{ T})^{1/2}$, respectively.

However, we stress that the exponential term $\exp(-\Delta_{LL}/2kT)$ depends both on temperature and field (due to the B-dependence of the LL gap Δ_{LL}). As shown in the Figure S6a, within 180 K and 300 K, the exponential results in a relative increase of the conductivity exceeding by more than one order of magnitude the predicted σ_T. As a consequence, the T-linear contribution is likely to remain elusive. Within 20 T and 30 T, instead, σ_B results in a relative variation of the conductivity in the same order of the exponential term (Figure S6d). Therefore, the $B^{1/2}$ dependence might be more realistically observed.

In Figure S6b we show the obtained σ_T as a function of T (with data points averaged over the different magnetic fields), compared to $\sigma_T = T/300$ K (dark cyan line). No temperature dependence can be conclusively identified from our data, in agreement with the scenario anticipated above. We note that similar fluctuations in the data points are also observed in the pre-factor to the data from Ref. [20] (Figure S6c). Hence, we conclude that the exponential term precludes the experimental assessment of a possible temperature dependence in the conductivity pre-factor.

In Figure S6e we show that σ_B (averaged over the different temperatures) follows the expected $B^{1/2}$ dependence. The quantitative agreement with the field dependence proposed in Ref. [24] supports the conclusions of our manuscript. In contrast, data from Ref. [20] are in accordance with a constant conductivity pre-factor (Figure S6f).

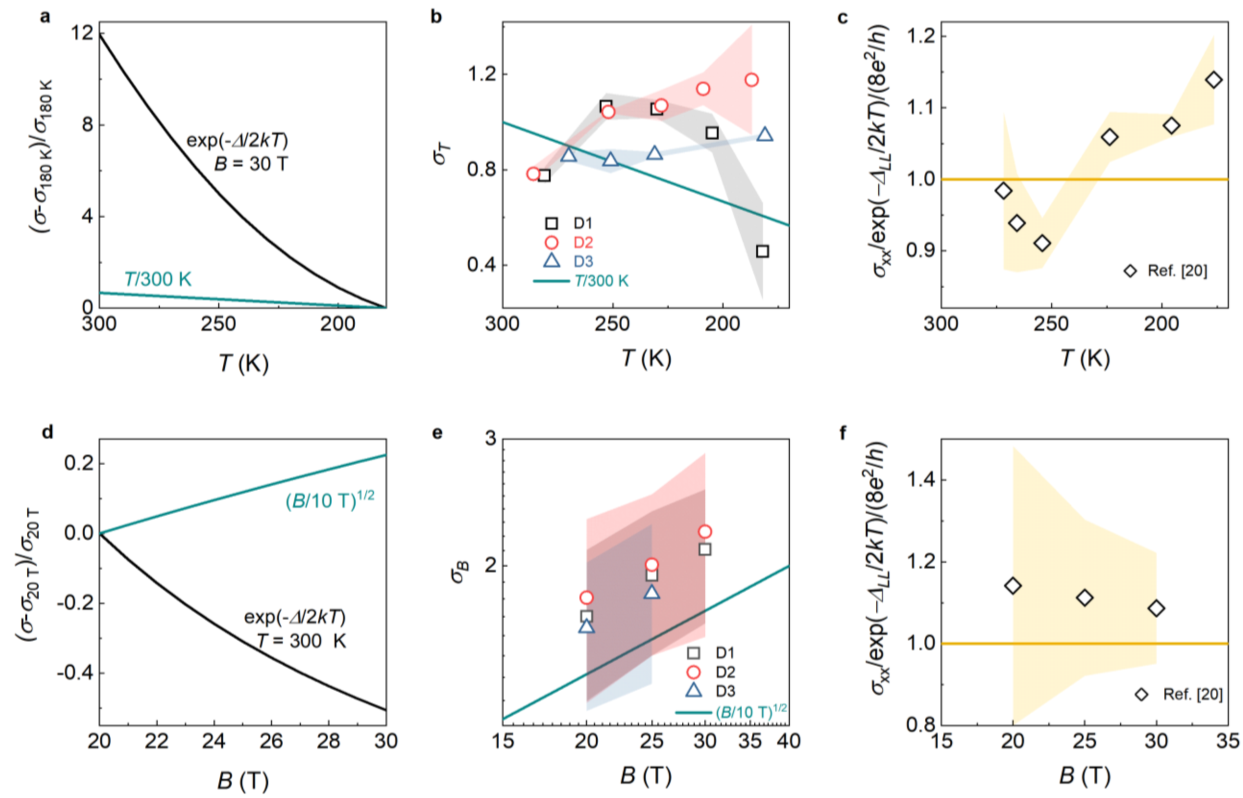

Figure S6 | Temperature and field dependence in the conductivity pre-factor. a, Relative variation of the conductivity over the range 180 K – 300 K, for the exponential term (black line) and the *T*-linear part of the pre-factor according to Ref. [24] (dark cyan line). **b,** Estimate of σ_T as a function of *T* for samples D1-3. The data points are obtained as $\sigma_T = [\sigma_{xx} / \exp(-\Delta_{LL}/2kT) - \sigma_D]/(\sigma_N\, \sigma_B)$, averaged over the different magnetic fields employed. The shaded areas correspond to ± one standard deviation. The dark cyan line is σ_T from Ref. [24]. **c**, Conductivity pre-factor as a function of *T*, for data from Ref. [20], normalized to $8e^2/h$, averaged over different fields. The shaded area is defined as in panel b. **d**, Relative variation of the conductivity over the range 20 T – 30 T, for the exponential term (black line) and the $B^{1/2}$ part of the pre-factor according to Ref. [24] (dark cyan line). **e,** Estimate of σ_B as a function of *B* for samples D1-3. The data points are obtained as $\sigma_B = [\sigma_{xx} / \exp(-\Delta_{LL}/2kT) - \sigma_D]/(\sigma_N\, \sigma_T)$, averaged over the different temperatures employed. The shaded areas are defined as in panel b. The dark cyan line is σ_B from Ref. [24]. The Log-Log scale is used to linearize the $B^{1/2}$ dependence. **f,** Conductivity pre-factor as a function of *B*, for data from Ref. [20], normalized to $8e^2/h$, averaged over different temperatures. The shaded area is defined as in panel b.

Supplementary Note 7: Temperature-driven interplay between disorder and phonon contributions

To gain a deeper insight on the interplay between disorder and phonon scattering in clean samples, we perform additional experiments at lower magnetic fields (produced by a standard superconducting coil) and, correspondingly, lower temperatures. We fabricate an additional device (D5), following the same methods of D1-3 (exfoliated graphene flakes). D5 shows transport characteristics fully comparable to D3 (at 220 K, we obtain $n^* \sim 4 \times 10^{10}$ cm^{-2} and carrier mobility ~1.4×10^5 cm^{2}V^{-1}s^{-1}), indicating that it is representative of the clean limit discussed in the main text.

We collect data at $B \geq 1$ T, to ensure full development of the QH effect at $\upsilon = 2$, as well as an activation energy matching $\Delta_{LL}/2$. At $B = 1$ T, we employ temperatures of 50 K to 100 K to observe activated behaviour (see Figure S7, panel a). According to Ref. [24], at $T = 100$ K e-ph scattering should result in a resistivity of ~100 Ω. Despite the high sample quality of D5, we measure values larger by more than one order of magnitude (~1.4 kΩ). This observation contrasts with the high-B high-T behaviour, where negligible deviation from the e-ph limit is measured for sample D3. By increasing the applied magnetic field and, accordingly, the temperature range, the activated resistivity drops, and the deviation from the e-ph limit tends to be suppressed (see panels b, c and d in Figure S7). We quantify the deviation at different magnetic fields and temperatures using the parameter ρ_D introduced in the main text, obtained by fits shown as black lines in Figure S7.

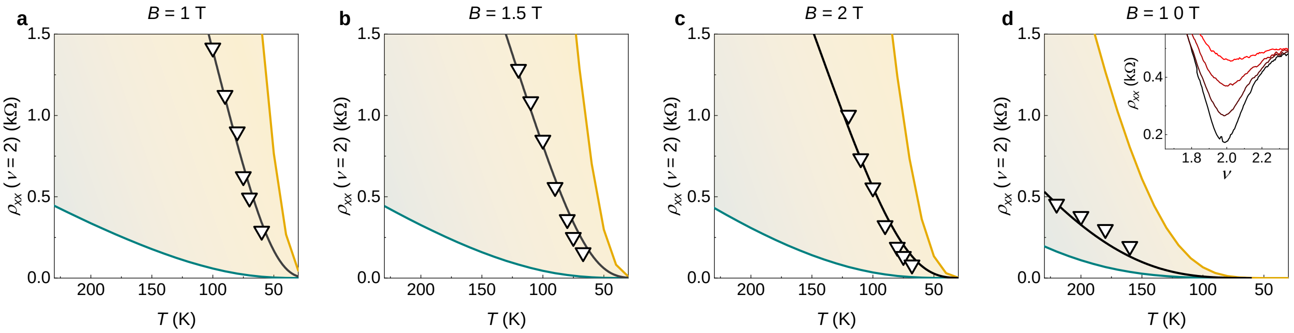

Figure S7 | Temperature-dependent resistivity at low magnetic field. a-d, Activated resistivity at filling factor 2 (black triangles), measured in sample D5 in a superconducting magnet for different magnetic fields. The data are compared to the e-ph and long-range disorder limits (dark cyan and yellow curves, respectively). The black

lines are fits to the activated resistivity, employed to extract the parameter ρ_D shown in Figure S8. The inset in panel d shows the raw resistivity data as a function of the filling factor at B = 10 T.

A more comprehensive picture can be proposed by combining the ρ_D parameters extracted for two samples of comparable quality, D5 and D3, as shown in Figure S8a. At B = 10 T, ρ_D drops by approximately a factor 2 with respect to 1 T. At $B \geq 20$ T, ρ_D vanishes (these are the same data points for D3 shown in main text Figure 4). The corresponding temperature ranges employed in the measurements are shown in Figure S8b. Throughout the experiments, the temperature ranges are adapted to the applied magnetic field, to follow the increase of the gap size and ensure that all the data are collected in comparable conditions of thermal activation. Overall, our results indicate that high-quality graphene devices can show both disorder and phonon-mediated contributions to the dissipative conductivity in the QH regime. The disorder contribution is non-universal, meaning that it is sample-dependent and tends to be suppressed with increasing temperature (and magnetic field). In clean enough samples, the e-ph contribution becomes dominant toward RT, where the QH effect is observable only at high magnetic fields ($B \geq 20$ T).

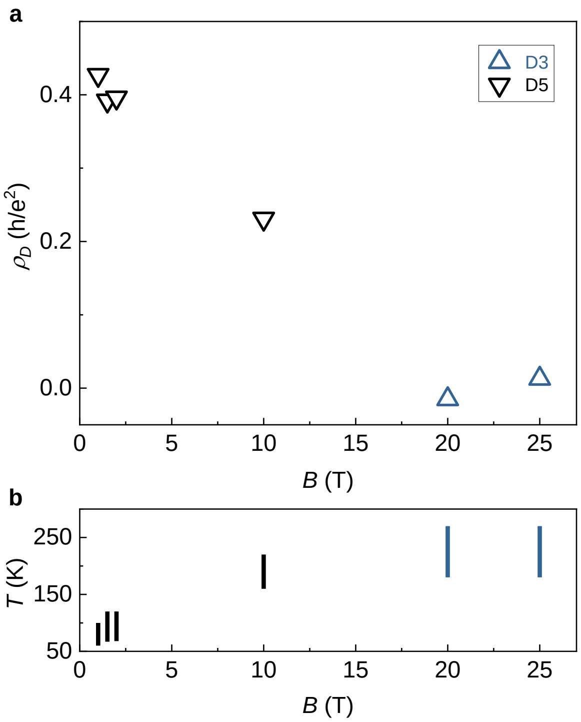

Figure S8 | Temperature-driven decay of the disorder contribution. a, Parameter ρ_D extracted for samples D3 and D5 at different magnetic fields. **b,** Temperature ranges employed for the measurements at the different magnetic fields.
